# Tumor Associated Stromal Cells Play a Critical Role on the Outcome of the Oncolytic Efficacy of Conditionally Replicative Adenoviruses

**DOI:** 10.1371/journal.pone.0005119

**Published:** 2009-04-08

**Authors:** M. Verónica Lopez, Diego L. Viale, Eduardo G. A. Cafferata, Alicia I. Bravo, Cecilia Carbone, David Gould, Yuti Chernajovsky, Osvaldo L. Podhajcer

**Affiliations:** 1 Laboratory of Molecular and Cellular Therapy, Instituto Leloir, IIBBA-CONICET, Ciudad Autónoma de Buenos Aires, Buenos Aires, Argentina; 2 Molecular Immunopathology Section, Hospital Eva Perón, Buenos Aires, Argentina; 3 Faculty of Veterinary Sciences, University of La Plata, La Plata, Argentina; 4 Bone and Joint Research Unit, Barts and the London, Queen Mary's School of Medicine and Dentistry, University of London, London, United Kingdom; University of Florida, United States of America

## Abstract

The clinical efficacy of conditionally replicative oncolytic adenoviruses (CRAd) is still limited by the inefficient infection of the tumor mass. Since tumor growth is essentially the result of a continuous cross-talk between malignant and tumor-associated stromal cells, targeting both cell compartments may profoundly influence viral efficacy. Therefore, we developed SPARC promoter-based CRAds since the SPARC gene is expressed both in malignant cells and in tumor-associated stromal cells. These CRAds, expressing or not the Herpes Simplex thymidine kinase gene (Ad-F512 and Ad(I)-F512-TK, respectively) exerted a lytic effect on a panel of human melanoma cells expressing SPARC; but they were completely attenuated in normal cells of different origins, including fresh melanocytes, regardless of whether cells expressed or not SPARC. Interestingly, both CRAds displayed cytotoxic activity on SPARC positive-transformed human microendothelial HMEC-1 cells and WI-38 fetal fibroblasts. Both CRAds were therapeutically effective on SPARC positive-human melanoma tumors growing in nude mice but exhibited restricted efficacy in the presence of co-administered HMEC-1 or WI-38 cells. Conversely, co-administration of HMEC-1 cells enhanced the oncolytic efficacy of Ad(I)-F512-TK on SPARC-negative MIA PaCa-2 pancreatic cancer cells *in vivo*. Moreover, conditioned media produced by stromal cells pre-infected with the CRAds enhanced the *in vitro* viral oncolytic activity on pancreatic cancer cells, but not on melanoma cells. The whole data indicate that stromal cells might play an important role on the outcome of the oncolytic efficacy of conditionally replicative adenoviruses.

## Introduction

The concept that the stromal microenvironment is a crucial regulator of tumor development was originally proposed by Paget many years ago in his “*seed and soil*” hypothesis [1]. Essentially, tumors are heterogeneous organs that in addition to the malignant cells contain cancer-associated fibroblasts, endothelial and inflammatory cells [1], [2]. These tumor-companion cells are intermingled in the tumor-associated stroma that comprises most of the tumor mass in many carcinomas and provide the soil in which malignant cells will grow, invade and metastasize [3]–[5]. Malignant and stromal cells communicate one to each other through cell-cell and cell-matrix interactions and secretion of soluble factors, providing an intratumor connection that is essential for tumors growing beyond a certain size [5]. Despite this evidence, only recently research in cancer turn to the tumor environment aiming to establish how malignant and stromal cells communicate and “coconspirate’ in tumor development [6].

Conditionally replicative adenoviruses (CRAd) are engineered to selectively replicate within and kill tumor cells through the use of “cancer cell”-selective promoter elements that transcriptionally restrict expression of genes essential for CRAd replication [7]. Different CRAds had recently reached the clinic although they did not realize the expectations regarding their potential therapeutic effectiveness [8]. Different strategies were applied to improve CRAds efficacy including viral retargeting through the exchange of the capsid fiber or addition of specific moieties such as the RGD that will retarget vectors to enter the cell through cell surface integrins [9], [10].

Despite those efforts, clinical CRAds efficacy is still limited and the reasons for that remain elusive, although viral spread or “lateralization” appears as one of the most important aspects. Stromal cells -especially fibroblasts- can impose limitations for lateral spread because of their physical presence and their capacity to produce extracellular matrix that might affect CRAd activity and interaction with malignant cells. In this regard, CRAd limited efficacy might arise in part from the fact that they are generally designed to target the malignant cell compartment alone while no attention was paid on neighbor stromal cells. Only very recently efforts were aimed to combine CRAd activity with enzymatic degradation of the stroma [11] with the potential risk of releasing tumor cells to the circulation. However, no evidence was presented yet on the role stromal cells might play in defining CRAd activity. Since stromal cells “cross talk” with malignant cells can define tumor outcome it can be hypothesized that, unless simultaneously targeted, they will severely limit CRAd replication and spreading.

SPARC (Secreted Protein Acidic and Rich in Cysteines) has been associated with a family of non-structurally related proteins like Tenascin and Thrombospondin that regulate cell adhesion by inducing focal cell contacts disassembly [12]. SPARC, also named Osteonectin, is a secreted glycoprotein whose expression in healthy tissues is largely associated with tissue remodeling, morphogenesis, and wound healing [13]. In adults, its expression is greatly diminished and restricted to areas with certain levels of cellular turnover such as gut epithelia and central lens epithelia, Leydig cells and Sertoli cells [13]–[15]. SPARC is produced by different cell types including malignant cells and tumor associated stromal cells such as fibroblast and endothelial cells [16]. It was found to bind to different components of the extracellular matrix such as collagens, and to interact with specific growth factors or with their signaling pathways such as VEGF, PDGF and bFGF, suggesting that it might play a role in vasculogenesis [17]. Among the many biological processes modulated by SPARC, its anti-adhesive [18], [19], pro-migratory [20], and anti-apoptotic [21] properties on certain cell types in addition to its capacity to regulate matrix metalloproteinase (MMP) expression and activity [17], [22], [23] have been associated with increased tumor agresiveness. Indeed, increased SPARC expression has been described in multiple cancers, including colon [24], esophagus [25], pancreas [26], breast [27], lung [28], brain [29], bladder [30], renal [31], and melanoma [18], [32]. SPARC overexpression by the malignant cells themselves or by neighbor fibroblasts and endothelial cells has been associated with poor prognosis in different human cancer types [16], [33], [34]. Primary and metastatic melanoma samples expressed high levels of SPARC in malignant cells and in intermingled fibroblasts and endothelial cells, whereas dysplastic nevi, benign nevi and normal melanocytes exhibited low to none SPARC expression [32]. Moreover, SPARC expression by peritumoral fibroblasts portends a poorer prognosis for patients with pancreatic cancer [33].

Reports characterizing the 5′ region of the SPARC gene, including the non-translated exon 1, revealed a significant homology between the murine, bovine and human gene, including the lack of canonical TATA or CAAT box sequences and the presence of a purine-rich stretch composed of two boxes, named GGA-box 1 and 2 separated by a 10 bp pyrimidine-rich spacer element [35]. The GGA1 box provides maximal promoter activity, and deletion of the spacer element appears to increase the activity induced by the GGA boxes [35]. Interestingly, the region comprising approximately 1.4 Kb that includes both GGA boxes was suggested to be essential for cell-type specific regulation of the bovine promoter but not of the human promoter [36]. This difference might arise from a GC-box with multiple Sp1 binding sites located upstream of the GGA-boxes in the bovine promoter which is not conserved in the human promoter [36]. More recent data observed with the human promoter demonstrated the presence of an non-canonical AP1-binding site between −120 bp and −70 bp that can bind a c-Jun/Fra1 heterodimer *in vitro* [37]. This c-Jun responsive element is an SP-1 binding site and appears to be sufficient to induce maximal promoter activation [37]. We have recently shown that a 1.3 Kb SPARC promoter fragment was effective in driving the expression of the Herpes Simplex virus thymidine kinase gene (TK) both in melanoma and endothelial cells leading to the elimination of melanoma tumors *in vivo* in nude mice [38]. These data led us to hypothesize that SPARC promoter could be a good candidate for generating a CRAd to target the malignant and stromal cell components of the tumor mass that will be strongly attenuated in normal, non-cancer associated cells.

Here, we show that the oncolytic efficacy of these novel CRAds depends on the specific interactions that the malignant cells establish with neighbor stromal cells. This interaction might restrict or augment CRAds efficacy depending on the tumor type. We also propose that the design of CRAds should consider stromal cells as a potential target for achieving improved efficacy.

## Results

### Selection of a SPARC promoter fragment to drive E1A gene expression

**Figure 1:**
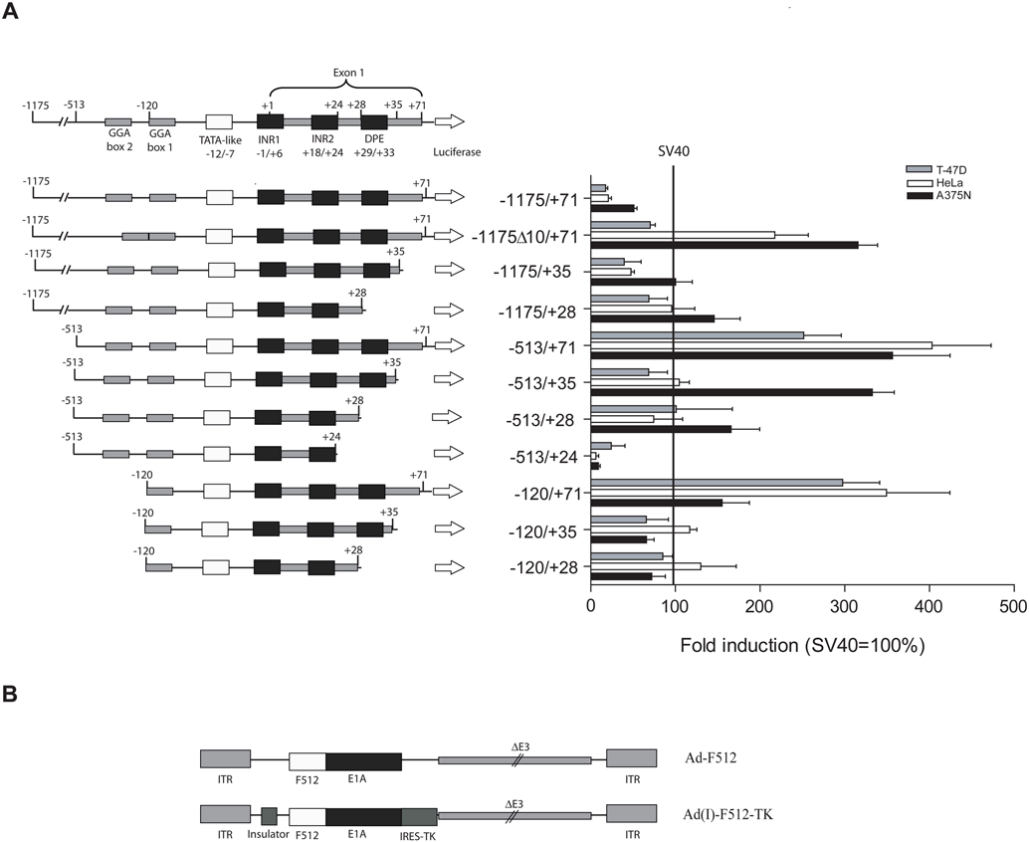
Luciferase activity driven by different variants of SPARC promoter. (A) Left: the different variants of SPARC promoter. Right: Luciferase activity of the different variants of SPARC promoter different cells lines. Data are shown relative to SV40 luciferase activity. Error bars represent mean±SD. (B) Schematic structure of Ad-F512 and Ad(I)-F512-TK genomes.

In order to target both the tumor and stroma compartment we designed a CRAd based on the SPARC promoter since SPARC was shown to be expressed both in malignant and tumor-associated stromal cells [16]. We first assessed the activity of different promoter fragments that were generated maintaining the integrity of specific motives such as two GGA boxes that confer maximal activity, a TATA-like box, two transcription initiation sites [35] and a putative downstream promoter element (DPE [39]). Promoter activity was assessed by cloning each fragment into the promoterless firefly luciferase reporter plasmid pGL3-Basic followed by cell transfection and luminescence quantification. By comparing luciferase levels in A375N melanoma cells that overexpress SPARC compared to breast cancer T-47D and cervical cancer HeLa cell lines that exhibited very low levels of SPARC (see Table 1 for relative SPARC mRNA levels in different cell lines), we selected the −513/+35 fragment, and named it F512Pr (Figure 1A). F512Pr showed 3.3-fold higher activity than the SV40 promoter in A375N cells, while in HeLa and T-47D has similar or less activity than the SV40 promoter (Figure 1A). F512Pr exhibited the highest luciferase activity (1.7 to 4.9 - fold induction over SV40 promoter) in melanoma cell lines that do express high SPARC mRNA levels although no strict relationship was observed between SPARC mRNA levels and promoter activity (Figure S1A and Table 1). We were unable to efficiently transfect additional non-malignant stromal cells such as WI-38 fibroblasts and HMEC-1 endothelial cells (data not shown).

**Table 1:** Relative expression of SPARC mRNA levels in different malignant and non-malignant cell lines.

Cell Line	Source	Relative Expression^a^	n^b^
A375N	Melanoma	100.00	3
MEL888	Melanoma	15.23±0.61	3
MEL-J-N	Melanoma	23.65±2.97	3
SB2	Melanoma	31.20±8.11	3
IIB-MEL-LES	Melanoma	7.36±0.58	3
BxPC3	Pancreas cancer	0.00±0.00	2
MIA PaCa-2	Pancreas cancer	0.00±0.00	3
T-47D	Breast cancer	0.15±0.04	3
MCF-7	Breast cancer	0.00±0.00	2
MDA-231	Breast cancer	0.00±0.00	2
NHM	Normal melanocytes	8.00±4.42	2
CCD841	Normal colon	4.03±1.12	3
MCF12-A	Normal breast	0.00±0.00	2
HMEC-1	Human endothelium	20.23±1.21	4
HaCaT	Keratinocyte	0.05±0.05	2
CCD1140	Fibroblast	41.70±6.09	4
Malme-3	Fibroblast	78.00±4.60	2
WI-38	Fibroblast	19.47±1.67	3

a The data was obtained by real time – PCR and is expressed as the relative expression with respect to the levels observed in the A375N cell line. The numbers correspond to the mean±standard deviation.

b Number of replicates.

### CRAds cytopathic effect on malignant cells

Since stromal cells were hardly transfectable and the activity of the promoter might change in the context of a viral structure, we initially constructed a non-replicative adenoviral vector (Ad(I)-F512-luc) where luciferase activity was driven by the F512Pr. All the cell types tested, including fibroblasts and endothelial cells exhibited promoter activity; in some cases higher than those observed in malignant cells (Figure S1B). Based on this preliminary evidence we constructed two new CRAds based on F512Pr. Ad-F512 contained F512Pr upstream of the E1A gene (Figure 1B). Ad(I)-F512-TK contained an insulator element [40] that isolated F512Pr-E1A from the enhancer elements located in the left ITR, and the TK suicide gene that was placed downstream of E1A following an IRES sequence (Figure 1B).

**Figure 2:**
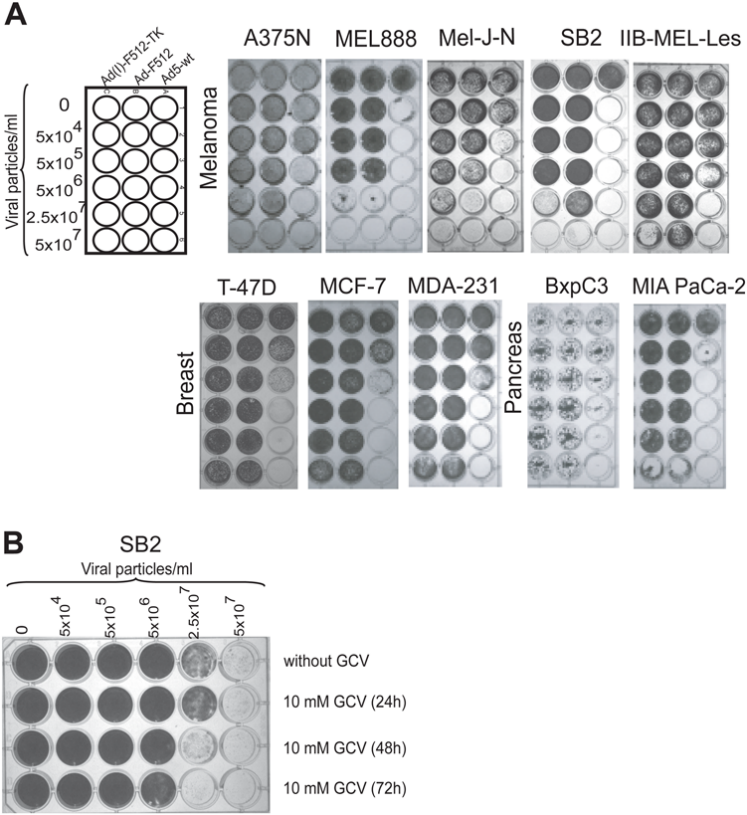
CRAd lytic activity *in vitro* on human cancer cells. (A) Cell viability after infection with Ad-wt, Ad-F512, or Ad(I)-F512-TK. Human cancer cell lines were infected at different concentrations of the CRAds. (B) Viability of SB2 melanoma cells after infection with Ad-(I)F512-TK in the presence or not of GCV added at the indicated times. Remaining cells were fixed and stained with crystal violet at day 10.

The cytopathic effect (CPE) of the two CRAds was compared on a panel of tumor cells that expressed or not SPARC mRNA. Different human malignant cell lines were infected with different concentrations of the CRAds or adenovirus wild type (Ad-wt) that was used as a control for viral replication and lytic effect that was assessed by crystal violet staining followed by densitometer quantification. Initial experiments were performed in the absence of GCV to test whether addition of the TK gene could be deleterious to CRAd activity. Ad-wt exhibited CPE on almost every cancer cell line at a low titer of 5×10^4^ (MOI of 1) to 5×10^6^ vp/ml (MOI of 100) regardless of SPARC expression levels (Figures 2A and S2A). On the contrary, both CRAds exhibited CPE mainly on SPARC positive-human melanoma cells at relatively moderate viral concentrations of 5×10^6^ to 2.5×10^7^ vp/ml (Figures 2A and S2A). It was of note that high concentrations of the CRAds exerted a lytic effect on SPARC-negative MIA PaCa-2 cells (Figure 2A and Table 1). By using E4 production as readout of viral replication we confirmed that the CRAd replicated not only in SB2 melanoma cells but also in MIA PaCa-2 pancreatic cancer cells (Figure S3). The importance of CRAd replication in SPARC-negative cells is described below. In general, Ad(I)-F512-TK exhibited increased CPE compared to Ad-F512 and addition of GCV, not before 48 hr after viral infection, enhanced its lytic activity indicating that the TK gene was active (Figures 2B and S2B).

### Lack of CRAds cytopathic effect on normal cells

**Figure 3:**
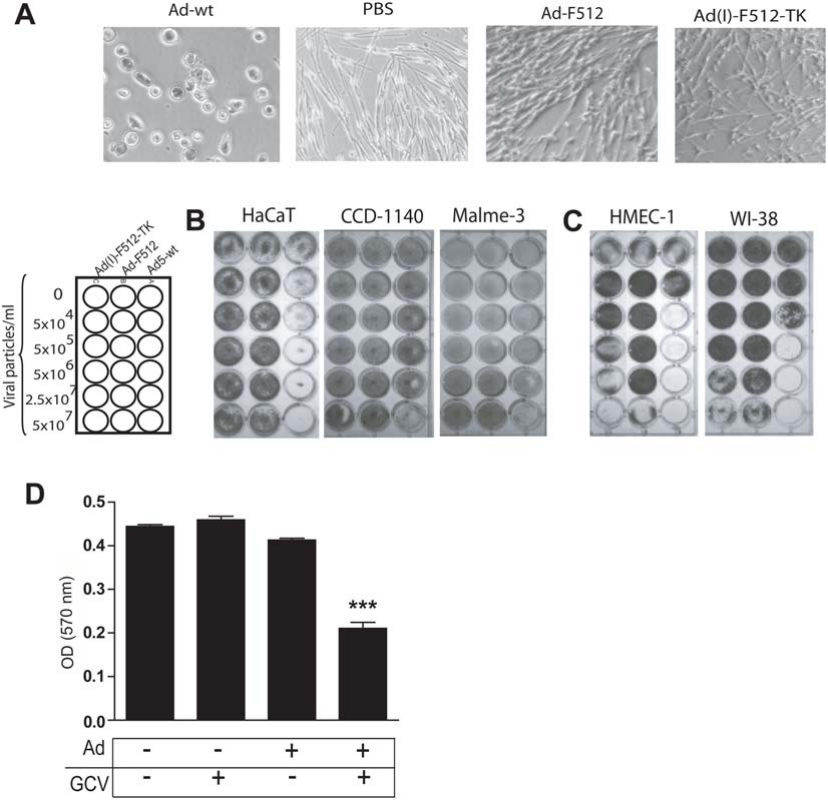
Assesment of CRAd lytic activity *in vitro* on non-cancerous human cells. (A) Lysis of normal melanocytes by Ad5-wt but not by the CRAd. (B) HaCaT adult kerantinocytes and normal adult fibroblasts sensitivity to Ad-wt but not to the CRAds. (C) Lytic activity of the Adwt and the CRAd on WI-38 fibroblasts and HMEC-1 cells (Of note is that HMEC-1 cells detached from the well in the absence of virus). (D) Reduced HMEC-1 viability after infection with Ad-(I)F512-TK (5×10^6^ vp/ml) followed by GCV. Remaining viable cells were stained as described. ***P<0.001 (one-way ANOVA follow by a Tukey multiple comparison test).

We next established the level of attenuation of Ad-F512 and Ad(I)-F512-TK lytic activity on normal cells. By day 10 following infection with 5×10^7^ vp/ml of Ad-F512 or Ad(I)-F512-TK, the viability of normal melanocytes was >95% compared to complete melanocytes elimination by Ad-wt (Figure 3A). Similarly, Ad-F512 and Ad(I)-F512-TK exhibited no CPE on CCD841 normal colonic cells or normal MCF12A breast cells, whilst Ad-wt lysed these cells at 5×10^6^ vp/ml (Figures S4A and B). Moreover, HaCaT keratinocytes were sensitive to the lytic effect of Ad-wt while they were completely refractory to the lytic activity of F512Pr-based CRAds (Figure 3B and S4C). Finally, the SPARC-positive CCD-1140 and Malme-3 normal adult skin fibroblasts were sensitive to the highest Ad-wt concentration but were completely resistant to Ad-F512 or Ad(I)-F512-TK probably due to the absence of E1B region in the present CRAd constructs [41] (Figure 3B and S4C). These data demonstrate that normal cells are completely resistant to the F512Pr-based CRAds regardless of whether they expressed SPARC or not.

### CRAds cytopathic effect on transformed endothelial cells and fetal fibroblasts

We next assessed CRAd's CPE on SPARC-positive large T antigen-transformed microendothelial cells (HMEC-1) and WI-38-fetal lung fibroblasts that could be considered as resembling the potential characteristics of cancer-associated stromal cells. Both cell types were lysed by Ad(I)-F512-TK in the absence of GCV (Figures 3C and S4C) although WI-38 cells were at least one order less sensitive (Figure 3C). The higher sensitivity of HMEC-1 cells was probably the result of their higher transducibility compared to WI-38 fibroblasts as assessed through the use of a non-replicative adenovirus expressing luciferase (Figure S5). In fact, both cell types were slightly permissive to viral replication compared to malignant cells (Figure S3). Addition of GCV improved Ad(I)-F512-TK lytic capacity on HMEC-1 cells, as assessed by the MTT assay (Figure 3D). Similar studies with WI-38 cells evidenced no improvement of CRAd activity (data not shown). Thus, SPARC-positive HMEC-1 endothelial cells supported viral replication and were sensitive to their cytotoxic activity.

### The presence of stromal cells restricted the therapeutic efficacy of the CRAds on established melanomas

**Figure 4:**
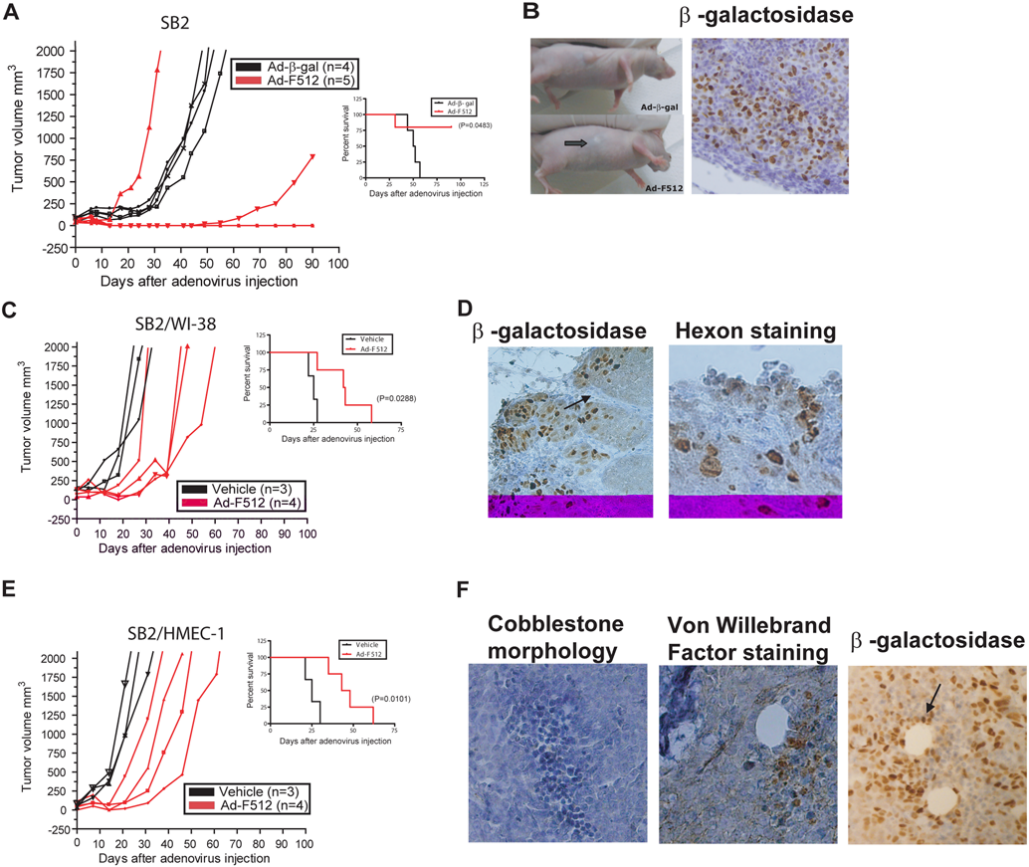
Ad-F512 effect on the *in vivo* growth of human melanoma xenografts. (A) Tumor growth in mice harboring SB2 melanomas treated with Ad-F512 or control virus. Right panel shows Kaplan-Meier survival curves. (B) Left panel includes photographs of an Ad-β-gal – treated mouse and a mouse treated with the CRAd. The arrow shows complete tumor regression, 14 days after Ad-F512 administration. (C) Tumor growth in mice harboring Mel/Fib tumors treated with Ad-F512 or control virus. Right panel shows Kaplan-Meier survival curves. (D) Left panel shows Mel/Fib tumors staining positive for β-galactosidase in the malignant nests but not in fibroblasts septa (arrow), and right panel shows adenoviral hexon- positive staining of Mel/Fib tumors. (E) Tumor growth in mice harboring Mel/Endo tumors treated with Ad-F512 or control virus. Right panel shows Kaplan-Meier survival curves. (F) Left panel includes nests of microendothelial cells with the typical cobblestone morphology; middle panel shows endothelial cells staining positive for Von Willebrand and right panel shows Mel/Endo tumors staining positive for β-galactosidase both in malignant cells and in endothelial cells lining intratumor vessels (arrow). In figures corresponding to the *in vivo* studies, each curve corresponds to a single animal.

Having established the lytic efficacy of the CRAds on the different cell types, we next decided to examine the ability of Ad-F512 to inhibit the growth of human melanoma xenografts s.c. established in nude mice following administration of malignant cells alone or mixed with stromal cells. Mice carrying established SB2 melanoma tumors were treated with three consecutive i.t. administrations at days 0, 3 and 7 of 10^10^ vp/mouse of Ad-F512, Ad-β-gal or vehicle alone. None of the mice benefited from the administration of Ad-β-gal or vehicle as all the tumors reached 2 cm^3^ when mice were considered not survivors (Figure 4A and Figure S6). Treatment with Ad-F512 resulted in a potent antitumor effect, as the tumor completely disappeared in 3 of 5 mice and one mouse exhibited reduced tumor growth (Figure 4A). One mouse remained alive for almost one year (data not shown). Another mouse exhibited accelerated tumor growth, most probably due to incomplete transduction and/or viral dissemination [42]. In two additional studies, 2/4 and 3/5 mice treated with Ad-F512 remained free of tumor at the end of the experiments at 90 days (Figures 6SA and B). Cured mice exhibited complete tumor remission 2–3 weeks after the last CRAd administration (Figure 4B) that was accompanied by a massive macrophage infiltrate probably associated with clearance of cellular debries (data not shown). Tumor injection with Ad-β-gal showed staining preferentially at the tumor periphery with a gradual decrease towards the inner part of the tumor (Figure 4B). Thus, a CRAd driven by the SPARC promoter was therapeutically effective leading to the cure of more than 50% of mice harboring human melanomas composed of malignant cells alone.

To better understand the role of stromal cells we next studied whether Ad-F512 could be therapeutically effective on established tumors composed of melanoma and stromal cells. Mice were xenografted with a mix of 4.0×10^6^ SB2 melanoma cells and 2.0×10^6^ WI-38 fetal fibroblasts (Mel/Fib tumors, Figure 4C). Mel/Fib tumor's architecture closely resembled human tumors with nests of malignant cells separated by septa of fibroblasts (Figure 4D) compared to the homogeneous appearance of tumors made of malignant cells alone (see Figure 4B, β-galactosidase). In the presence of WI-38 fibroblasts, Ad-F512 exerted a statistically significant delay on Mel/Fib tumor growth but no cure was observed (Figure 4C). Interestingly, only malignant cells at the peripheral nests, but almost no WI-38 fibroblasts, were infected, as evidenced by positive staining for β-gal activity (Figure 4D, β-galactosidase). Malignant cells also showed positive hexon staining after Ad-F512 administration, indicating viral replication (Figure 4D, hexon staining).

We further examined the role of stromal cells by xenografting mice with a mix of 4.0×10^6^ SB2 cells and 2×10^6^ HMEC-1 cells (Mel/Endo tumors, Figure 4E). Nests of HMEC-1 distributed all over the tumor mass exhibiting cobblestone morphology (Figure 4F, cobblestone morphology), and few of them that surrounded blood vessels stained positive for factor VIII indicating endothelial differentiation (Figure 4F, Von willebrand factor staining). Interestingly, both melanoma and endothelial cells surrounding blood vessels stained positive for virus infection following administration of Ad-β-gal (Figure 4F, β-galactosidase). Thus, despite the absence of a clear physical barrier for viral spreading in Mel/Endo tumors, the presence of endothelial cells impaired Ad-F512 activity as WI-38 did, and no cure was observed.

**Figure 5:**
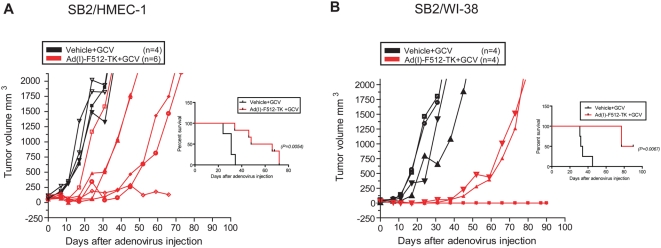
Ad(I)-F512-TK+GCV effect on the *in vivo* growth of human melanoma xenografts including stromal cells. (A) Tumor growth and Kaplan-Meier survival curve of mice harboring Mel/Endo tumors, treated with Ad(I)-F512-TK or vehicle, followed by GCV. (B) Tumor growth and Kaplan-Meier survival curve of mice harboring Mel/Fibro tumors treated with Ad(I)-F512-TK or vehicle followed by GCV. Each curve represents a single animal.

Given the reduced therapeutic efficacy of Ad-F512 in the presence of stromal cells, we sought to establish whether TK expression combined with GCV might help to overcome tumor resistance due to the presence of stromal cells in the tumor mass. Thus, mice harboring Mel/Endo tumors were treated with Ad(I)-F512-TK followed by 2 weeks of GCV administration. GCV started to be administered 4 days after the last viral injection to facilitate maximal viral spreading and to avoid cell death induced by GCV before the virus started replicating. Under these conditions, 5 out of 6 mice showed tumor growth delay after treatment with Ad-(I)F512-TK and GCV including one tumor that ceased growing compared to none in control mice (Figure 5A). Similar experiments performed on established Mel/Fib tumors showed inhibition of growth with Ad-(I)F512-TK/GCV in all mice compared to the control, and complete remission in 2 mice (Figure 5B). Thus, Ad-(I)F512-TK/GCV demonstrated improved efficacy compared to Ad-F512 since few cures were observed after treatment, although as a whole, the presence of stromal cells hampered viral lytic efficacy.

### The presence of stromal cells improved the therapeutic efficacy of the CRAd on established pancreatic cancer

Figure 2A showed that the CRAds exerted a lytic effect on MIA PaCa-2 cells despite the absence of SPARC expression. Previous studies have shown that SPARC promoter is hypermethylated in pancreatic cancer cells [26]. That should not necessarily affect the activity of an extrinsic promoter carried by the CRAd. Indeed F512Pr was able to drive luciferase expression in the context of an adenoviral vector in MIA PaCa-2 cells, although at a lower level compared to A375N melanoma cells (Figure S7). Based on that, we assessed whether the present CRAd may be active on SPARC-negative pancreatic cancer cells *in vivo* in the presence or not of co-administered stromal cells.

**Figure 6:**
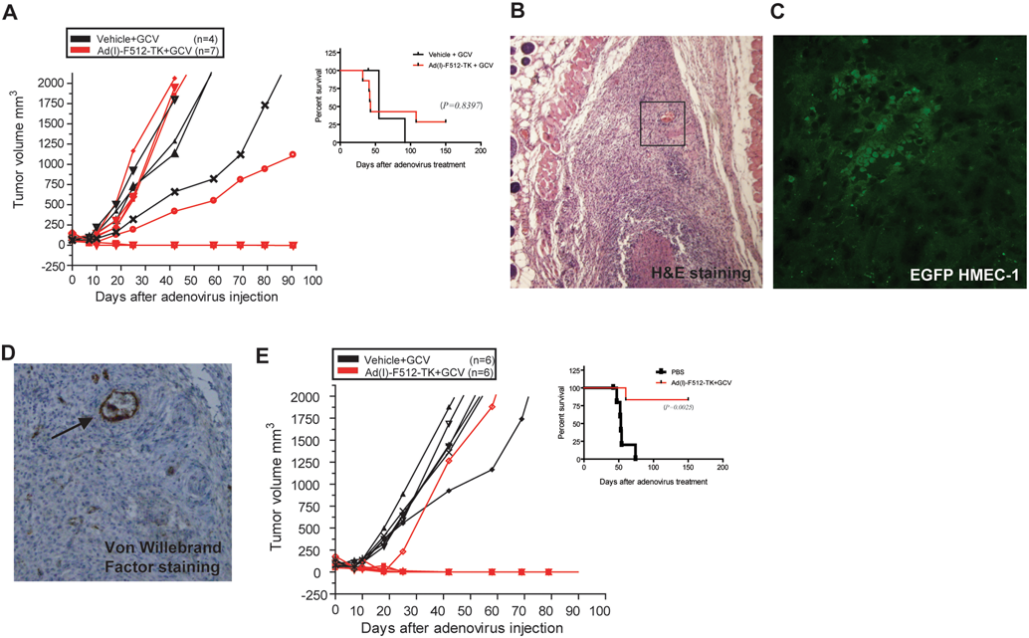
Ad(I)-F512-TK+GCV effect on the *in vivo* growth of human pancreatic cancer xenografts. (A) Tumor growth and Kaplan-Meier survival curve of mice harboring MIA PaCa-2 tumors, treated with Ad(I)-F512-TK or vehicle, followed by GCV. (B) Hematoxylin-eosin staining of Pan/Endo tumors; the area limited by the square is enlarged in D. (C) Typical nests of microendothelial cells (EGFP-HMEC-1) with cobblestone morphology in a Pan/Endo tumor. (D) Large and small vessels (arrow) staining positive for Von Willebrand factor. (E) Tumor growth and Kaplan-Meier survival curve of mice harboring Pan/Endo tumors, treated with Ad(I)-F512-TK or vehicle, followed by GCV. Each curve represents a single mouse. Error bars represent mean±SD.

Treatment of established MIA PaCa-2 tumors made of malignant cells alone with Ad(I)-F512-TK+GCV induced remission of 2 out of 7 tumors while the other 5 grew as the controls although this difference was not statistically significant (Figure 6A). This suggests that SPARC promoter was partially active on pancreatic cancer despite the lack of SPARC expression. We next treated mice harboring tumors made of an initial mix of 9.0×10^6^ MIA PaCa-2 cells and 2.0×10^6^ HMEC-1 cells (Pan/Endo tumors). We selected HMEC-1 cells over WI-38 fibroblasts based on their higher infectivity *in vitro* and *in vivo* and their capacity to inhibit the *in vitro* growth of a co-culture of HMEC-1: MIA PaCa-2 cells when HMEC-1 cells were pre-infected with Ad(I)-F512-TK in the presence of GCV (Figure S8).

Pan/Endo tumors showed loosely packed malignant cells surrounded by a dense extracellular matrix that was independent of the presence or not of HMEC-1 cells (Figure 6B). Pancreatic tumors obtained without endothelial cells showed a similar morphology (data not shown). In order to confirm that HMEC-1 cells were present in the tumor mass at the time of CRAd infection we pre-infected them with a lentivirus expressing GFP. Microscopic observation of tumor slices confirmed the presence of EGFP-positive cells that in some cases formed typical cobblestone structures (Figure 6C), and tend to form vessels that often stained positive for factor VIII (Figure 6D). Ad(I)-F512-TK administration followed by GCV induced complete tumor remission in all but one mice (5/6) strongly indicating that the presence of HMEC-1 microendothelial cells in the pancreatic tumor mass favored the therapeutic efficacy of Ad-(I)-F512-TK/GCV (Figure 6E).

### Soluble factors produced by stromal cells greatly influence viral oncolytic efficacy

**Figure 7:**
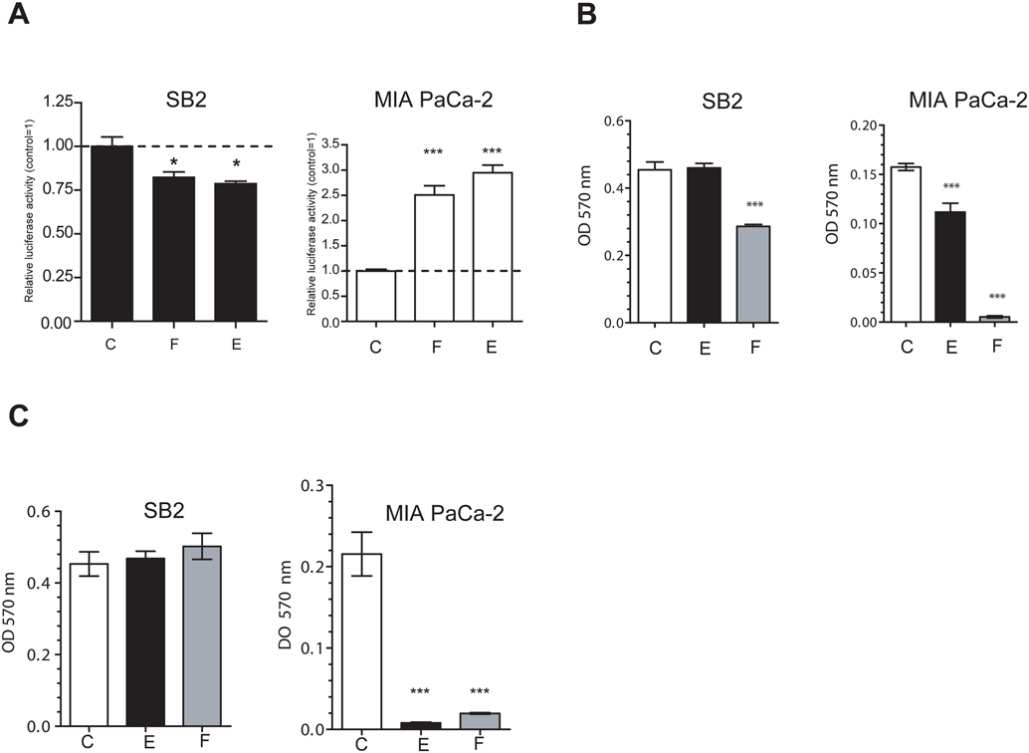
Effect of conditioned media produced by stromal cells on CRAd activity. (A) Transcriptional activity of F512Pr in SB2 and MIA PaCa-2 cells following cell infection with Ad(I)-F512-luc in the presence of HMEC-1 (column E), WI-38 (column F)- or their own conditioned medium (column C). (B) Cell viability after infection of SB2 and MIA PaCa-2 cells with Ad(I)-F512-TK in the presence of the different conditioned media. (C). Cell viability after infection of SB2 and MIA PaCa-2 cells with Ad(I)-F512-TK in the presence of the different conditioned media obtained from previously infected cells. *P<0.05, ***P<0.001 (one-way ANOVA follow by a Tukey multiple comparison test).

The striking differences in CRAd activity observed between melanoma and pancreatic cancer when microendothelial cells were co-administered with malignant cells and the evidence that microendothelial cells and fibroblasts hampered CRAd activity irrespective of tumor architecture, led us to hypothesize that soluble factors might have been playing a role. WI-38- and HMEC-1-conditioned media induced a slight inhibition of F512Pr activity in SB2 melanoma cells (Figure 7A). In clear contrast, both WI-38 and HMEC-1 conditioned media strongly enhanced F512Pr activity in pancreatic MIA PaCa-2 cancer cells (Figure 7A). In addition, WI-38-conditioned media enhanced viral lytic activity on SB2 cells and other melanoma cells as well (Figure 7B and S9A). Moreover, both conditioned media enhanced at a different extent CRAd activity on MIA PaCa-2 cells (Figures 7B and S9A).

Surprisingly, preinfection of stromal cells with the CRAd completely obliterated the enhancement in CRAd lytic activity induced by the conditioned media on SB2 melanoma cells (Figure 7C). But in clear contrast, conditioned media obtained from pre-infected HMEC-1 and WI-38 cells dramatically enhanced CRAd lytic activity on MIA PaCa-2 cells (Figure 7C). This effect was observed even at 1/5000 dilution indicating its potency (Figure S9B). Thus, soluble factors produced by stromal cells can play a dramatic and differential role in defining the therapeutic efficacy of a CRAd on specific tumor types.

### Soluble factors produced by stromal cells enhance pancreatic cancer cells exit from quiescence

**Figure 8:**
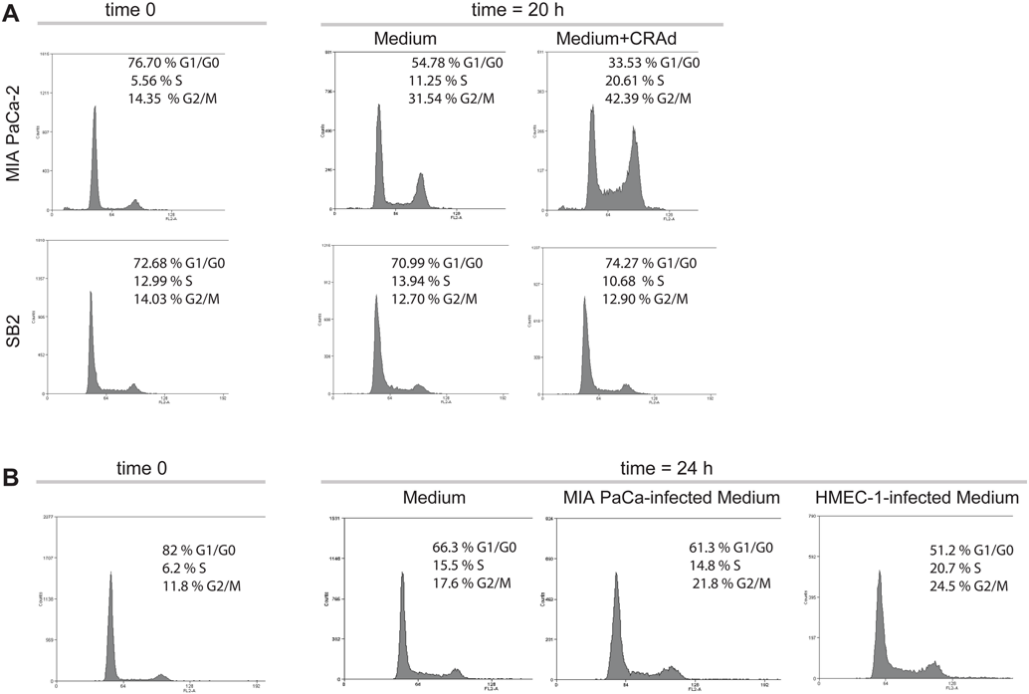
Effect of conditioned media produced by stromal cells on cell cycle. (A) Exit from cell cycle quiescence of MIA PaCa-2 and SB2 cells pre-infected with the CRAd. (B) Exit from cell cycle quiescence of MIA PaCa-2 cells in the presence of different conditioned media. One biological replicate out of two is shown in each case.

Previous evidence demonstrated that oncolytic viruses increased the amount of cells in S-phase [43] and that E1B mutant viruses, such as the present CRAds, exhibited better cytopathic effect on cells in S phase [44]. To dissect CRAd's effects, we first assessed whether arrested malignant cells infected with the CRAd showed an accelerated exit from quiescence compared to non-infected cells. Indeed, infected MIA PaCa-2 cells exhibited a higher cell number in S-phase at 20 hr compared to non-infected cells (Figure 8A). Surprisingly, SB2 melanoma cells exhibited a retarded exit from G0/G1 compared to MIA PaCa-2 cells, regardless of whether SB2 cells were infected or not with the CRAd (Figure 8A).

Next, we assessed whether stromal cells-conditioned media might also accelerate MIA PaCa-2 cells exit from quiescence. Twenty four hours after MIA PaCa-2 cells release from G0/G1, we observed a clear increase in the amount of cells in S-phase when they were exposed to conditioned media obtained from HMEC-1 cells pre-infected with the CRAd (Figure 8B), compared to MIA PaCa-2-own conditioned media obtained from cells infected also with the CRAd (Figure 8B) or the control media (Figure 8B).

## Discussion

This work provides the first evidence on the key role that stromal cells play in the outcome of the oncolytic efficacy of conditionally replicative adenoviruses. To our surprise the presence of stromal cells was detrimental for CRAd efficacy on melanoma tumors but favored CRAd oncolytic activity on pancreatic tumors. Soluble factors secreted by stromal cells were responsible for at least two potential mechanisms that might explain this differential efficacy: the levels of viral lytic activity and the timing for cell exit from quiescence. Thus, either because they hampered or enhanced CRAd activity, stromal cells might play an important role in defining the maximal oncolytic capacity of these viruses.

We designed conditionally replicative adenoviruses based on a specific segment of the SPARC promoter with the aim to target both malignant and tumor-associated stromal cells. Either CRAd, expressing or not TK were cytotoxic *in vitro* on a panel of melanoma cell lines expressing SPARC. Mostly important, normal cells from diverse sources were completely refractory to the CRAds while under the same conditions they were eliminated by wild type Ad5. Of note, Ad-F512 and Ad(I)-F512-TK were unable to lyse normal adult fibroblasts while exhibited lytic effect on cells that resemble cancer-associated stromal cells such as transformed microendothelial cells and fetal fibroblasts.

We observed a clear therapeutic efficacy of our CRAds on melanoma tumors made of malignant cells alone. Although the CRAd expressing TK appeared to exhibit a slightly enhanced therapeutic efficacy both CRAds exhibited diminished efficacy when tumors contained in addition stromal cells. Co-administration of WI-38 fibroblasts imposed to melanoma tumors an architecture consisting of malignant cell nests separated by fibroblasts septa that appeared as a physical barrier for the CRAds and were refractory to viral infectivity *in vivo*. Previous studies suggested that the presence of physical barriers produced by stromal cells or their increased production of extracellular matrices imposed restrictions for viral spread and hence, diminished viral therapeutic efficacy [42], [45]. These physical barriers also appeared to restrict viral infection to malignant cells in close proximity to blood vessels [46]. Therefore, co-administration of proteolytic enzymes that degrade the ECM or the use of vasoactive compounds were proposed as alternatives to improve the therapeutic efficacy of oncolytic viruses [11], [47] with the risk of releasing malignant cells to the circulation. While it is likely that the presence of WI-38 cells could hamper viral spread, it was surprising that the presence of HMEC-1 also restricted viral efficacy although no histological evidence of a physical barrier was observed. On the contrary, soluble factors secreted by WI-38 fibroblasts, but not HMEC-1 endothelial cells, exerted a slight but significant enhancement of CRAd activity on melanoma cells *in vitro* that was obliterated when the conditioned media was obtained from pre-infected WI-38 cells. In this regard it was of interest that Ad(I)-F512-TK+GCV showed better efficacy on Mel-Fibro than on Mel-Endo tumors, suggesting that soluble factors produced by non-infected WI-38 cells might have enhanced CRAd activity *in vivo*. Although highly unlikely, we cannot rule out that host cells could have contributed in some aspects to the results since the F512 human promoter is active in rodent fibroblasts *in vitro* (data not shown).

It was not unexpected that the present CRAds were also slightly effective on SPARC-negative pancreatic cancer cells. Previous evidence has shown that promoters carried by the CRAd are not subjected to the transcriptional regulations imposed to endogenous promoters such as hypermethylation [48]. More striking was the evidence that co-administration of endothelial cells enhanced the therapeutic efficacy of the CRAd in pancreatic tumors. This was consistent with the fact that soluble factors produced by fibroblasts and microendothelial cells enhanced viral activity in pancreatic cancer cells, especially when conditioned media were obtained from pre-infected cells. Previous evidence from the literature have demonstrated that oncolytic viruses increased the amount of cells in S-phase [43]. Even more important, E1B mutant viruses, such as the present CRAds, exhibited better cytopathic effect on cells in S phase [44]. The present work indicates that the CRAd accelerates pancreatic cancer cells exit from quiescence and induces the release of soluble factors produced by stromal cells that enhance the number of pancreatic cancer cells in S-phase. Both effects might converge in an enhanced lytic effect compared to melanoma cells. Thus, despite the fact that adenoviruses are known for their capacity to infect cycling and non-cycling cells, our data suggest that non-cycling cells could avoid CRAd attack. This is especially important since a consensus exists that potential cancer stem cells are not cycling under steady state conditions. Thus, the identification of the soluble factors produced by stromal cells that can accelerate G0/G1 exit of malignant cells could be very useful to design improved vectors or as potential adjuvant of virotherapy. In preliminary experiments using neutralizing antibodies we identified the mitogenic factor bFGF as one the products present in stromal cells-conditioned media, supporting the idea that accelerated release of malignant cells from quiescence could be used as adjuvancy. In this regard, recent evidence demonstrate that lentiviral vectors that, similar to adenoviruses, were supposed to infect both cycling and quiescent cells appear to be more effective in the presence of mitogenic factors such as EGF [49] supporting the idea that the induction of cell reentry from quiescence could provide a better scenario to enhance CRAd effectiveness.

The potential clinical use of an oncolytic vector based on SPARC promoter raised the question as to whether downregulation of SPARC expression might serve as an escape mechanism for a cell to avoid oncolytic attack. The role of SPARC in tumor progression is controversial although in certain types of human cancer such as glioblastoma and melanoma, SPARC overexpression in the malignant cells themselves has been associated with increased aggressiveness ([16] and references therein). Interestingly, SPARC overexpression by tumor-associated fibroblasts in pancreatic and lung cancer is also a marker of bad prognosis despite the fact that the malignant cells themselves are SPARC-negative due to promoter hypermethylation [33], [50]. Based on the present data we might hypothesize that if cell clones that are SPARC-negative due to promoter hypermethylation emerge due to the selective pressure imposed by CRAd attack, they might be still susceptible to the treatment if the DNA binding proteins that regulate SPARC promoter activity are being expressed. Alternatively, malignant SPARC-negative cells such as those found in pancreatic cancer might be susceptible to oncolytic attack if they are surrounded by stromal SPARC-positive cells. In this regard, a very recent article also raised the possibility to overcome the barrier imposed by cancer associated fibroblasts by using selected mutant viruses with enhanced capacity to replicate in cancer associated stromal cells [51]. Indeed, multiple passages *in vivo* of a wild type adenovirus 5 in a human pancreatic tumor xenografted in nude mice selected a mutant virus with enhanced antitumor activity and augmented lytic effect and progeny release both in malignant cells and in cancer associated fibroblasts [51].

Adenoviral retargeting through partial or complete modification of the fiber, has become a method of choice to improve viral efficacy [52]. We observed that melanoma/stroma xenografts grew faster than xenografts of melanoma cells alone (see control groups in Figure 4) indicating the need for improved viral infectivity since human tumors are a heterogeneous mix of malignant and stromal cells. It has been shown that stable melanoma cell lines express CAR, while short term cultures of primary melanomas very often lack CAR expression [52]. Primary melanomas were very efficiently transduced by other adenovirus serotypes or by pseudotyped particles with chimeric or genetically modified fibers [52]–[54]. However, tumor endothelium appears to be very efficiently infected by adenovirus type 5 [55]. Thus, deeper knowledge of the *in vivo* expression of viral receptors in the different cellular compartments of the tumor mass (malignant and stromal cells) might help improve the therapeutic efficacy of virotherapy.

Stromal cells are a major component of human cancer tissue and “cross talk” with malignant cells to stimulate tumor growth and metastatic dissemination. We believe that their co-administration in murine models of cancer is essential to better understand viral spread and oncolytic efficacy. As we show here, they can support viral replication and modulate the efficacy of a CRAd through the production of soluble factors. Testing additional CRAds that target only malignant cells or using non-permissive stromal cells in mixed models as the one described in this article, would be useful to establish whether targeting stromal cells can definitely improve the *in vivo* antitumor efficacy of oncolytic vectors.

## Materials and Methods

### Cell Culture

The human cell lines HeLa (cervical cancer, CCL-2), T-47D (breast cancer, HTB-133), MDA-MB-231 (breast cancer, HTB-26), WI-38 (fetal lung fibroblasts, CCL-75), MCF-7 (breast cancer, HTB-22), MIA PaCa-2 (pancreas carcinoma, CRL-1420), BxPC-3 (pancreas adenocarcinoma CRL-1687), Malme 3 (normal skin fibroblast, HTB-102), CCD-1140 (normal skin fibroblast, CRL-2714), CCD841 (normal colon, CRL-1790), MCF12A (normal breast, CRL-10782), and 293HEK (human embryonic kidney, CRL-1573) were obtained from ATCC (American Tissue Culture Collection, Rockville, MD, USA). The HMEC-1 cells (Human Microvascular Endothelial Cells) were kindly provided by Francisco Candal (Atlanta, USA). The human melanoma cell line IIB-MEL-LES was already described [18]; MEL-J-N is an *in vitro* selected clone of IIB-MEL-J [18]; A375N, MEL888 and SB2 human melanoma cell lines and human normal melanocytes were kindly provided by Estela Medrano (Houston, USA). Keratinocytes (HaCaT) were kindly provided by Fernando Larcher (Madrid, Spain). All the cell lines were grown in the recommended medium supplemented with 10% of fetal bovine serum (Natocor, Cordoba, Argentina), 2 mM glutamine, 100 U/ml penicillin and 100 µg/ml streptomycin and maintained in a 37°C atmosphere containing 5% CO_2_. MIA PaCa-2 cells were also supplemented with 2.5% of horse serum (Invitrogen, Carlsbad, CA).

### Plasmids and recombinant adenoviruses

pGEM-hSPPr plasmid containing the −1175/+71 SPARC promoter [38] was used as a template to obtain the SPARC promoter variants displayed in Figure 1. Table 1S provides the list of primers used for the different clonings: the −1175/+35 promoter fragment was cloned using primers SPfse and R35; the −1175/+28 promoter fragment using primers SPfse and R28, the −513/+71 promoter fragment using primers F513 and R71, the −513/+35 promoter fragment using primers F513 and R35, the −513/+28 promoter fragment using primers F513 and R28 (Table S1), the −513/+24 promoter fragment using primers F513 and R24, the −120/+71 promoter fragment using primers F120 and Spas, the −120/+35 promoter fragment using primers F120 and R35, and the −120/+28 promoter fragment using primers F120 and R28. The hSPPrΔ10 promoter variant (1236 bp) was already described [38]. The different promoter variants were cloned in pCR4-TOPO (Invitrogen, Carlsbad, CA) and sub-cloned in the promoterless firefly luciferase reporter plasmid pGL3-Basic (Promega Corp., Madison, WI).

In order to improve the shuttle vector-pADPSY capacity [56], we replaced the RSV promoter with a multiple cloning site (MCS: *Spe*I, *Bcl*I, *Kpn*I, *Nhe*I, *Mlu*I, *Bgl*II, *Eco*RV, *Cla*I, *SnaB*I, *Sal*I) to create the pAd-Xp shuttle vector. Next, a fragment of 234 bp corresponding to the stop codon region of the bovine growth hormone gene [40] was PCR-cloned in the *Spe*I/*Kpn*I sites downstream of the ITR in the MCS to create a new shuttle vector, pAd-I-Xp (using primers INSU-F-SpeI: INSU-R-KpnI, Table S1). A *Bgl*II/*BamH*I-E1A gene fragment (+560/+1632 of the adenoviral genome) was subcloned in the *Bgl*II site of pAd-Xp or pAd-I-Xp followed F512 promoter cloning upstream of E1A in *Mlu*I/*Bgl*II sites, to obtain the shuttle plasmids pAd-F512-E1A or pAd-I-F512-E1A. The HSV-tk gene was amplified from the plasmid phSPPr-TK ([38]) and cloned in *Nco*I/*Sal*I sites of pCite-1 vector (Novagene, Madison, WI.). The fragment IRES-HSV-tk was extracted with *Eco*RI and *Sal*I, the *EcoR*I site was filled in and cloned in pAd-I-F512-E1A in the *Eco*RV/*Sal*I sites downstream of E1A to obtain the pAd(I)-F512-TK plasmid.

In order to produce pAd-SV40-luc vector the SV40-luciferase fragment was extracted from pGL3 with *Bgl*II/*Bam*HI and cloned in the *Bgl*II site of pAd-XP. pAd(I)-F512-luc was constructed in two steps. First, the luciferase gene was obtained from pGL3-513/+35 with *Bgl*II/*Bam*HI and cloned in the *Bgl*II site of pAd-XP vector to produce pAd-XP-luc. Next, the (I)-F512 sequence was obtained from pAd-I-F512-E1A with *Fsp*I/*Bgl*II and cloned into the *Fsp*I/*Bgl*II site of pAd-XP-luc to obtain pAd(I)-F512-luc. Finally, the 2256 bp fragment of CMV-Renilla was obtained from pRL-CMV (Promega Corp. Madison, WI) with *Bgl*II/*Bam*HI and subcloned in the *Bgl*II site of pAd-XP vector to create pAd-CMV-Renilla. To construct Ad-F512, Ad(I)-F512-TK, Ad(I)-F512-luc, Ad-CMV-Renilla and Ad-SV40-luc, the co-transfection in 293HEK cells was used [57]. Adenovirus amplification and purification were performed as described [58]. Physical particle concentration (viral particles/ml) was determined by absorbance at 260 nm. Determination of 50% tissue culture infective dose-TC ID_50_ was determined by standard plaque assay on 293HEK cells [58]. All the constructs and viruses were confirmed by restriction pattern and automatic DNA sequencing (ABI PRISM 377 DNA Sequencer, Applied Biosystems, Foster City, CA). Ad-wt was kindly provided by Dr Andre Lieber (Seattle, USA).

### Luciferase Assays and Real Time-PCR

Luciferase assay using plasmids were performed as described [38]. For assays using adenovirus, 5×10^4^ cells/well (seeded in 24-well plates) were transduced with Ad-SV40-luc at 25×10^6^ vp/ml in 200 µl of DMEM/F12 containing 2% FBS. Eight hundred µl of fresh medium containing serum was added 2 hours later followed by cells harvesting 48 hours later. Quantification of luciferase activity followed manufacturer's recommendations (Promega Corp. Madison, WI). Luciferase activity was normalized by protein concentration in the cell lysate (Bio- Rad, Hercules, CA).

For assessing promoter activity in the presence of the different conditioned media, cells were seeded in 24-well plates at a density of 7×10^4^ cells/well. The next day, cells were infected with Ad(I)-F512-luc (35×10^6^ vp/ml, about 100 of MOI) and Ad-CMV-Renilla (3.5×10^6^ vp/ml, about 10 of MOI) in 200 µl of 2% DMEM/F12. After 2 hours of infection the medium was removed and 0.4 ml of serum-free conditioned medium was added. Cells were harvested 48 hours later and dual luciferase assay was performed following manufacturer's recommendations (Promega Corp. Madison, WI).

Real Time-PCR was performed as described [38].

### Cell killing assays

Cells were seeded in 24-well plates at a density of 1×10^4^ cells/well (HMEC-1 density was 5×10^4^ cells/well). The next day, cells were infected with the corresponding virus in 200 µl of DMEM/F12 containing 2% FBS. After 4 hours of infection 0.8 ml of fresh medium containing 10% FBS was added. Cytopathic effect was monitored by staining viable cells with crystal violet [58]. For evaluation of Ad(I)-F512-TK activity followed by GCV, cells were infected at 5×10^6^ pv/ml in 200 µl. Twenty four hours later, cells were mixed and seeded onto a 24-well plate with uninfected cells at density of 5×10^4^ cells/well. The next day, cells were treated with 50 µM of GCV. The medium with GCV was replaced each 48 hours. After 5 days of exposure to GCV. The number of surviving cells was determined by the MTT assay [59].

### One step growth curves in malignant and stromal cells

SB2, MIA-PaCa-2, HMEC-1 and WI-38 cells (2×10^4^ cells/well) were infected at 100 MOI of Ad(I)-F512-TK or inactivated Ad(I)-F512-TK (heated during 20 minutes at 90°C) in 200 µl of DMEM/F12 containing 2% FBS. After 4 hr of infection medium was removed, the cells were washed with PBS to remove uninternalized viruses, and 0.5 ml of fresh medium containing 10% FBS was added. Cells were collected at time 0, 48 hr, 72 hr and 96 hr later. DNA was extracted from cells using the Illustra tissue & cells genomicPrep Mini Spin kit (GE Healthcare, Buckinghamshire, United Kingdom) and E4 gene was measured by Q-Real Time PCR [60], [61]. Genomic DNA was subjected to Real-Time PCR in an iCycler iQ System (Bio-Rad Laboratories, Hercules, CA, USA). Each 25 µl reaction volume contained 1 unit Platinum® Taq DNA polymerase (Invitrogen, Carlsbad), 1× PCR Reaction Buffer (20 mM Tris–HCl, 21 pH 8.4, and 50 mM KCl), 1.5 mM Mg_2_Cl, 2.5 g BSA, 0.01% Glycerol, 0.4 µM of each specific primer targeting the E4 region (Ad5, nucleotides 33806–34074) (Table S1), 200 µM of dNTPs and 0.3×SYBR Green Solution. PCR conditions were set as follows: 150 seconds at 94°C and then 39 cycles of 45 seconds at 94°C, 30 seconds at 60°C and 30 seconds at 72°C. All the reactions were performed in triplicate. Analysis of data was carried out using the iCycler software (Bio-Rad Laboratories, Hercules, CA, USA) by comparing test sample to a standard. Standard Curves were generated by serial dilutions of 10^10^ copies of Adenoviral DNA in a solution of control cellular genomic DNA. Total E4 copies per sample were normalized with the amount of DNA present in each sample and reported as E4 copies/ng of DNA.

### *In vivo* studies

Five to six - weeks old female athymic N:NIH(S)-nu mice (Faculty of Veterinary, University of La Plata, Argentina) were s.c. injected either with tumor cells alone or with a mix of tumor cells and stromal cells. When the average tumor volume reached 100 mm^3^, mice were randomly separated into different treatments. The corresponding groups received three intra-tumoral injections on days 0, 3 and 7, either of Ad-F512 or Ad(I)-F512-TK containing 1×10^10^ vp/mouse. Control mice were injected either with Ad-β-gal or vehicle indistinctly since both had no effect on tumor growth. In the case of GCV treatment, four days after the last adenovirus injection a daily dose of 30 mg kg^−1^ of GCV (Cymevene, Roche) was administered for 15 consecutive days. Tumor volumes were estimated weekly from caliper measurements (volume = 0.5×(width)^2^×length). Mice were sacrificed when tumors reached an average of 2000 mm^3^ and mice were considered not survivors. None of the mice showed signs of wasting or other visible indications of toxicity. *In vivo* experiments followed institution guidelines, and all animals under study received food and water ad-libitum.

GFP/HMEC-1 cells were obtained by infection of the endothelial cells with a GFP-lentiviral virus (GenScripts Corp., Scotch Plains, NJ, USA). Cells (1×10^5^) were plated into each well of a six-well plate and after 4 h infected with a multiplicity of infection of 5 in the presence of 8 µg per mL of polybrene (Sigma, St Louis, Missouri). Cells were incubated with the virus for 15 hr, washed and examined for green fluorescent protein (GFP) expression at 48 hr. After 48 hr antibiotic selection was initiated and stably expressing GFP cells were selected.

For histology studies, samples were fixed in 10% neutral-buffered formalin before paraffin embedding and cutting of 5-µm sections. Alternatively, samples were fixed in 4% paraformaldehyde for 1 hour, cryopreserved overnight in 30% sucrose, embedded in tissue OCT, and stored at −20°C. Cryostat sections of 9 µm were mounted on gelatin-coated slides. After hydration slides were hematoxylin and eosin stained. For immunohistochemical studies we used a goat anti-adenoviral hexon protein antibody AB1056 (Chemicon International, Hampshire, UK), a rabbit anti-β-galatosidase antibody A-11132 (Molecular Probes, Eugene, OR, USA) and a rabbit anti-human Von Willebrand Factor (Dako, Germany) followed by a biotinylated donkey anti-goat antibody (Jackson ImmunoResearch, West Grove, USA) or biotinylated goat anti-rabbit antibody (Vector Laboratories, Burlingame, CA, USA), respectively. Biotinylated secondary antibodies were used in conjunction with the Vectastain ABC kit (Vector Laboratories, Burlingame, CA, USA) and finally the reaction was visualized with DAB (Dako, Germany). Slides were counterstained with hematoxylin and photographed in an Olympus BX60 microscope.

### Ethics statement

All animal procedures were performed according to the rules and standards of German animal law and the regulations for the use of laboratory animals of the National Institute of Health, USA. Animal experiments were approved by the Ethical Committee of the Institute Leloir Foundation.

### Preparation of conditioned media

Four million cells were seeded on 100 mm cell culture dishes. Twenty four hours later cells, infected or not with 500 MOI of the CRAd, were placed in DMEM/F12 containing 2% FBS. Two hours later, medium was replaced by DMEM/F12 without serum and conditioning was followed for additional 24 hours, centrifuged and stored at −80°C. The amount of virus in the conditioned media was determined by 50% tissue culture infective dose (TC ID_50_) in a standard plaque assay on 293HEK cells [58]. An average of 10^7^–10^8^ vp/ml was found in all preparation.

### Flow cytometry

Cells (2.5×10^5^/well MW6) were arrested in G0/G1 by serum starvation for 72 hours and then kept in 1 ml of DMEM/F12 containing 2% FBS for two hours. Cells were infected or not at 1000 of MOI in the same medium. Four hours later, 2 ml of serum-free conditioned medium was added and final serum concentration was adjusted to 5%. Cells were trypsinized at the indicated times, washed with PBS, fixed in 70% ethanol at 4°C overnight, washed again with PBS, resuspended in PBS-triton-X100, treated with RNase (Sigma Co, San Luis, MO) and stained with propidium iodide. Cell cycle status was analyzed using a FACSCalibur flow cytometer (Becton Dickinson, Oxford, United Kinngdom). Ten thousand cells were analyzed in each case.

### Statistical analysis

Survival rates were calculated with the Kaplan–Meier method and differences were evaluated by the log-rank test. Statistical difference between groups was determined by one-way analysis of variance follow by a Tukey multiple comparison test. A *P*-value of <0.05 was considered statistically significant. Data analysis was performed with the GraphPad Prism 4.0 (GraphPad Software, Inc., San Diego, CA).

## Supporting Information

Figure S1 Luciferase activity driven by F512Pr. (A) In a plasmid context. (B) In an adenovirus context. Transcriptional activity of F512Pr promoter in stromal and malignant cells following infection with Ad(I)-F512-luc/Ad-CMV-Renilla. Data are expressed in RLU (Firefly/Renilla). Error bars represent mean±SD.
(5.58 MB EPS)

Figure S2 Quantification of Crystal violet assays. Densitometric analysis of wells corresponding to figures 2A and 2B was performed by using the Image J program available at http://rsb.info.nih.gov/ij; and developed by Wayne Rasband, National Institutes of Health, Bethesda, MD. Error bars represent mean±SD.
(1.07 MB PDF)

Figure S3 One step growth curve and viral production. Time dependent increase of E4 gene copy was used as a readout of CRAd replication. (A) Cells infected with Ad(I)-F512-TK and (B) Cells infected with heat-inactivated Ad(I)-F512-TK.
(0.29 MB PDF)

Figure S4 Cytophathic effect and quantification of Crystal violet assays. (A) CPE of normal colon cells CCD841. (B) Cell viability of normal breast cells MCF12A. (C) Densitometric analysis of wells corresponding to Figures 3B and C was performed by using the Image J program available at http://rsb.info.nih.gov/ij; and developed by Wayne Rasband, National Institutes of Health, Bethesda, MD. Error bars represent mean±SD.
(0.98 MB PDF)

Figure S5 In vitro transduction of HMEC-1 and WI-38 cells with non-replicative adenovirus. WI-38 and HMEC-1 cells were transduced with 5×106 vp/ml of Ad-SV40-luc as described in Material and Methods. Cell extracts were assayed two days later for firefly luciferase activity and protein concentration. Error bars represent mean±SD.
(0.27 MB PDF)

Figure S6 Ad-F512 effect on the in vivo growth of human melanoma xenografts. (A) and (B) Tumor growth in mice harboring SB2 melanomas treated with Ad-F512, vehicle or control virus (Ad-b-gal). Right panel corresponds to Kaplan-Meier survival curve of the tumor growth graft.
(0.38 MB PDF)

Figure S7 Luciferase activity driven by F512Pr in melanoma and pancreatic cancer cells. Transcriptional activity of F512Pr promoter in MIA PaCa-2 and A375N cells following infection with Ad(I)-F512-luc/Ad-CMV-Renilla. Data are expressed relative to Ad-SV40-luc activity in each cell line.
(0.26 MB PDF)

Figure S8 Viability of co-cultures of HMEC-1 cells infected ex vivo with Ad(I)-F512-TK- and human MIA PaCa-2 cells followed by GCV. Only the 20∶80 ratio (HMEC-1:MIAPaCa-2) is shown. *P<0.05 (one-way ANOVA followed by a Tukey multiple comparison test).
(0.27 MB PDF)

Figure S9 Effect of conditioned media produced by stromal cells on CRAd activity. (A) Cell viability after infection of the different cell types with Ad(I)F512-TK in the presence of different dilutions of conditioned media obtained from HMEC-1 cells, WI-38 fibroblasts or their own (B) Similar to A, but the conditioned media was obtained from pre-infected cells. For further details see Figure 7. Cell viability was assessed at day 6 of infection by using MTT assay.
(0.37 MB PDF)

Table S1 Primers sequences. The table shows the sequence of primers used for the different clonings.
(0.04 MB DOC)
