# Supplementary figures and images for: Tumor Associated Stromal Cells Play a Critical Role on the Outcome of the Oncolytic Efficacy of Conditionally Replicative Adenoviruses
Source: PLoS One. 2009 Apr 8;4(4):e5119. doi: 10.1371/journal.pone.0005119 (PMC2663040; doi:10.1371/journal.pone.0005119)

**A**

**A375N**

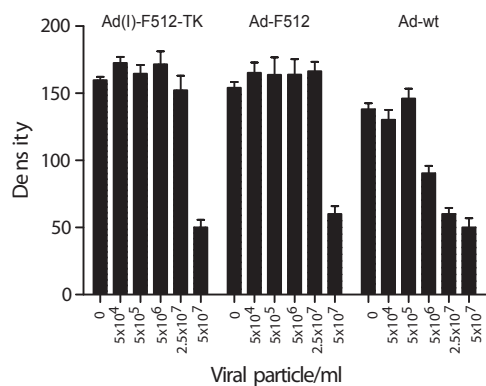

**MEL888**

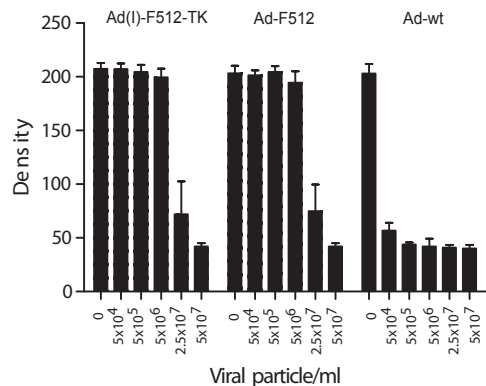

**Mel-J-N**

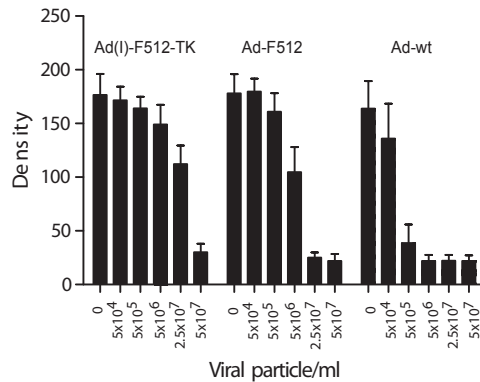

**SB2**

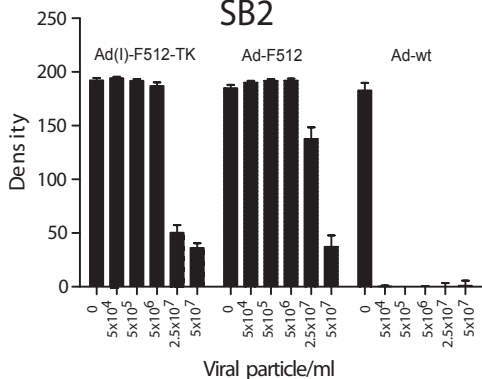

**IIB-MEL-Les**

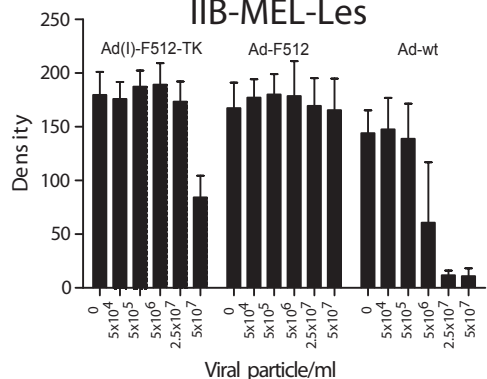

**T-47D**

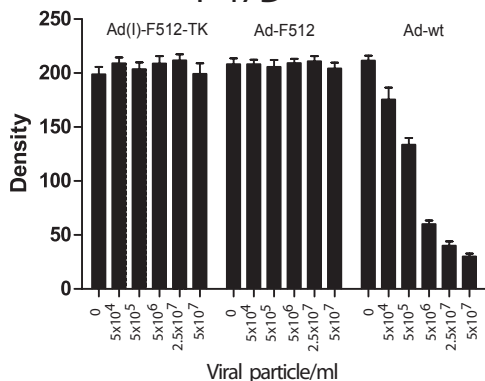

**MCF-7**

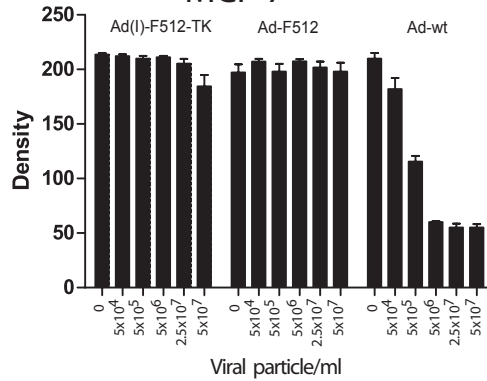

**MDA-231**

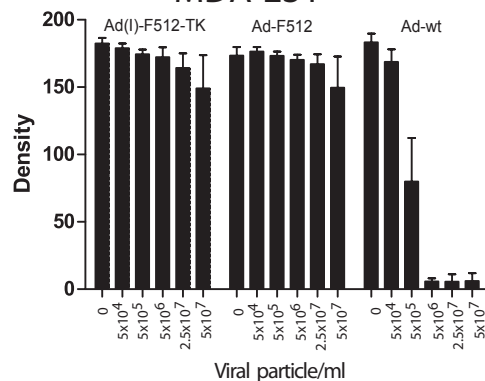

**BxpC3**

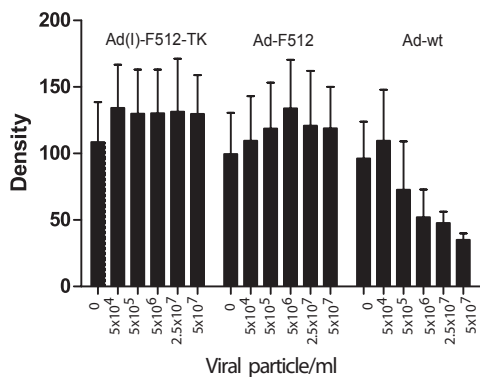

**MIA PaCa-2**

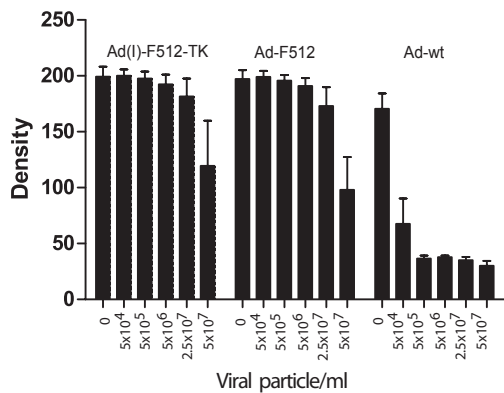

**B**

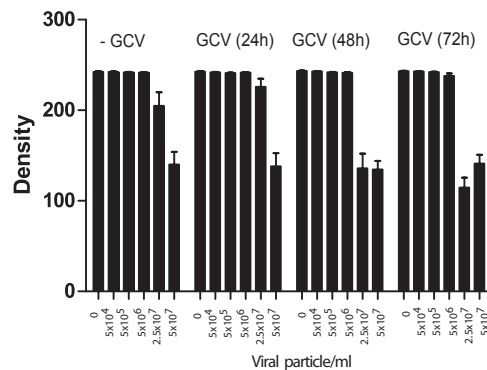

Supplement: Figure S2 — Quantification of Crystal violet assays. Densitometric analysis of wells corresponding to figures 2A and 2B was performed by using the Image J program available at http://rsb.info.nih.gov/ij; and developed by Wayne Rasband, National Institutes of Health, Bethesda, MD. Error bars represent mean±SD. (1.07 MB PDF) [file pone.0005119.s002.pdf]

**A**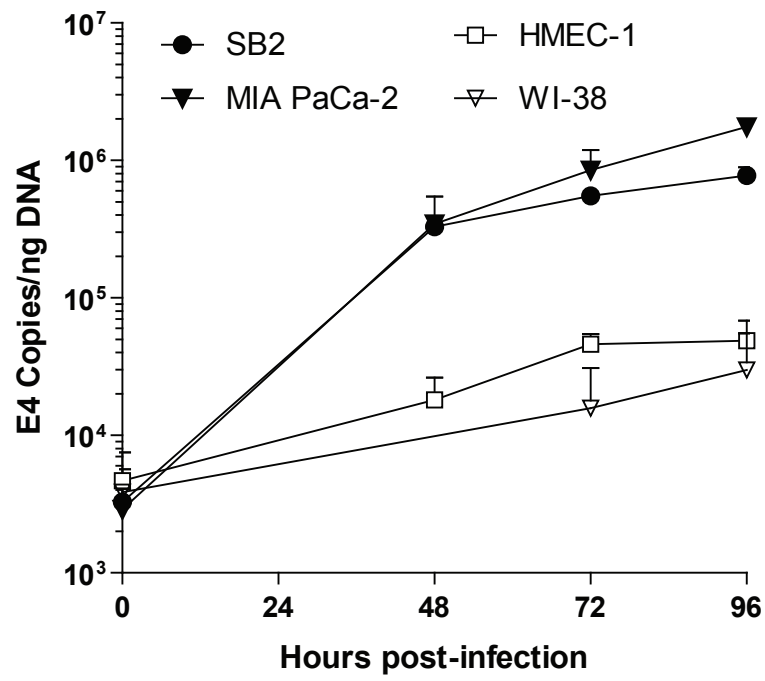**B**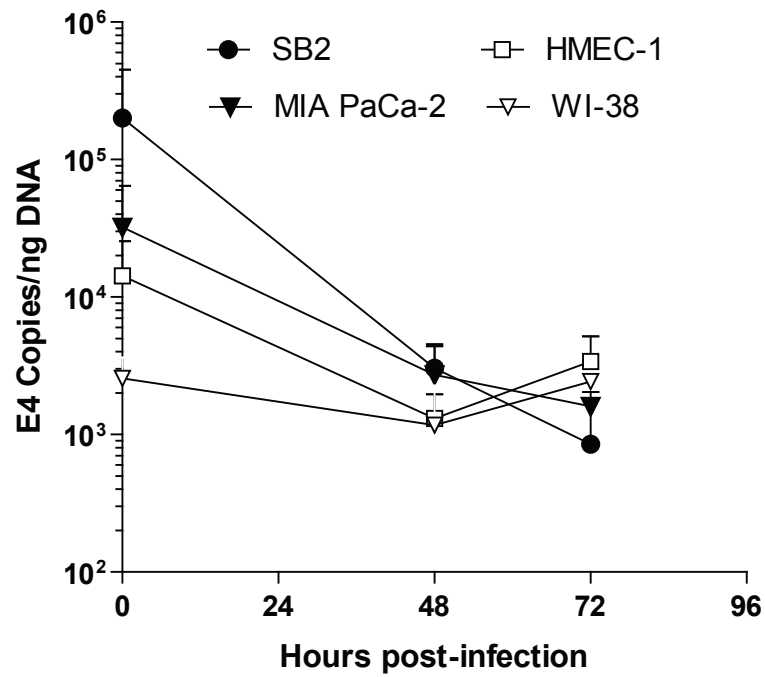

Supplement: Figure S3 — One step growth curve and viral production. Time dependent increase of E4 gene copy was used as a readout of CRAd replication. (A) Cells infected with Ad(I)-F512-TK and (B) Cells infected with heat-inactivated Ad(I)-F512-TK. (0.29 MB PDF) [file pone.0005119.s003.pdf]

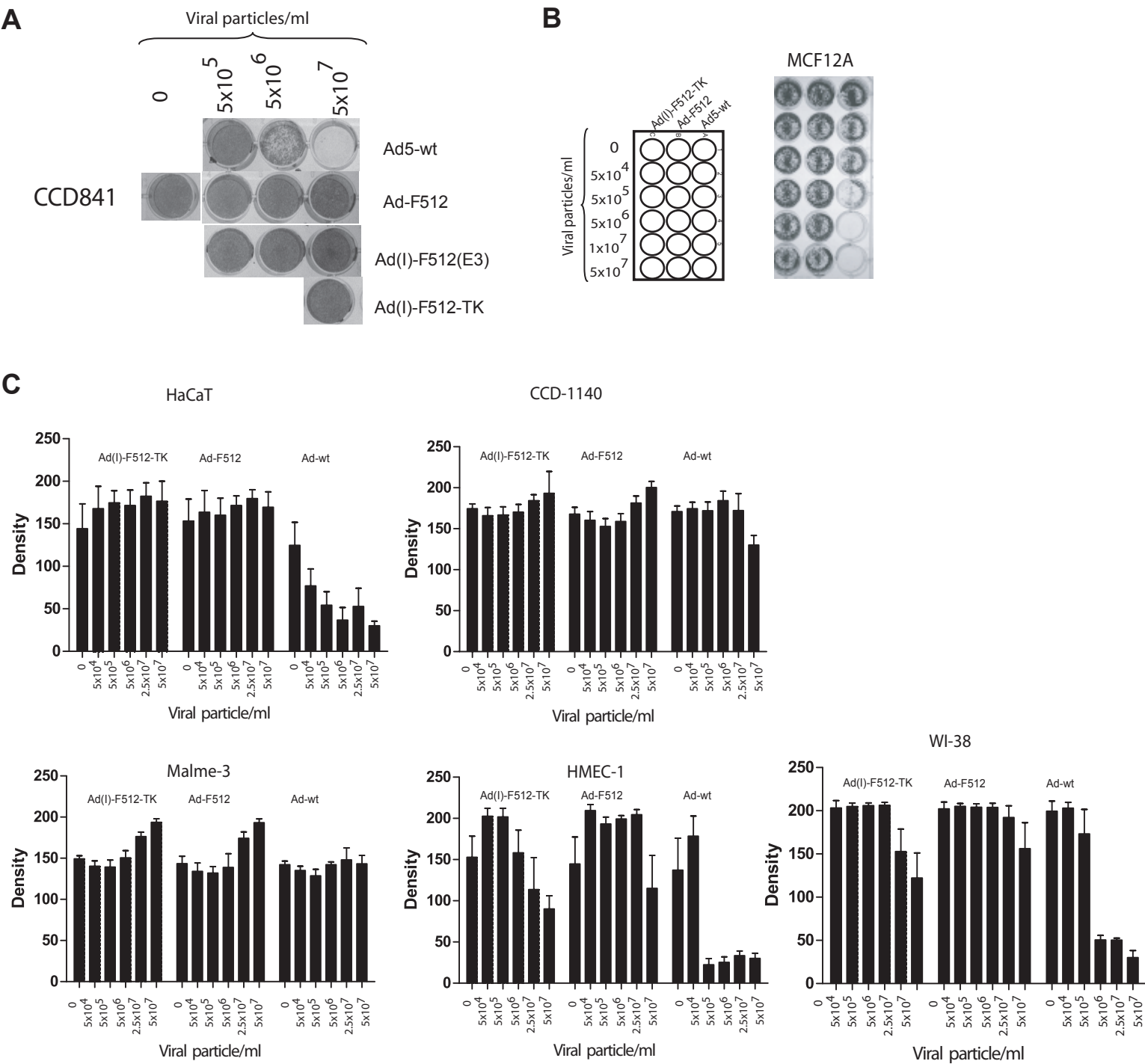

Supplement: Figure S4 — Cytophathic effect and quantification of Crystal violet assays. (A) CPE of normal colon cells CCD841. (B) Cell viability of normal breast cells MCF12A. (C) Densitometric analysis of wells corresponding to Figures 3B and C was performed by using the Image J program available at http://rsb.info.nih.gov/ij; and developed by Wayne Rasband, National Institutes of Health, Bethesda, MD. Error bars represent mean±SD. (0.98 MB PDF) [file pone.0005119.s004.pdf]

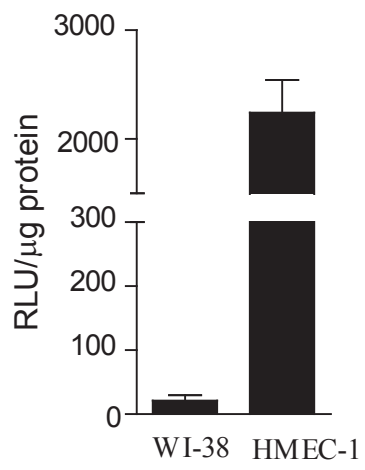

Supplement: Figure S5 — In vitro transduction of HMEC-1 and WI-38 cells with non-replicative adenovirus. WI-38 and HMEC-1 cells were transduced with 5×10^6^ vp/ml of Ad-SV40-luc as described in Material and Methods. Cell extracts were assayed two days later for firefly luciferase activity and protein concentration. Error bars represent mean±SD. (0.27 MB PDF) [file pone.0005119.s005.pdf]

**A**

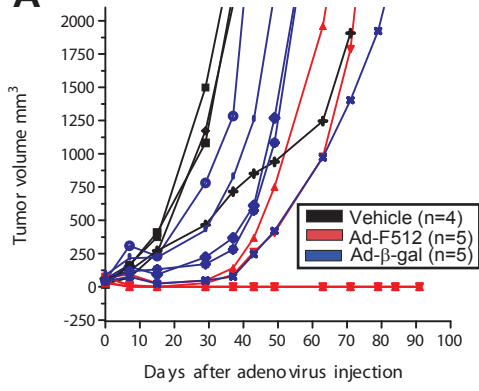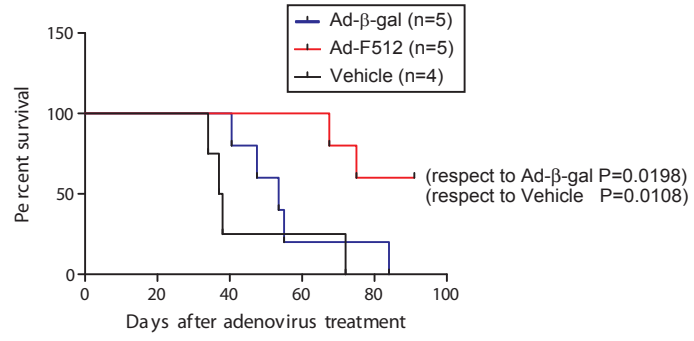

**B**

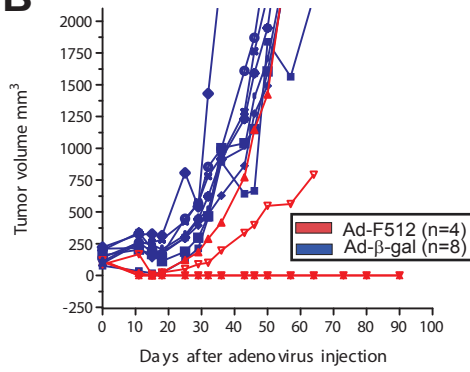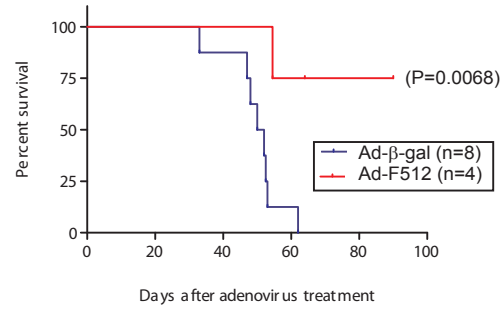

Supplement: Figure S6 — Ad-F512 effect on the in vivo growth of human melanoma xenografts. (A) and (B) Tumor growth in mice harboring SB2 melanomas treated with Ad-F512, vehicle or control virus (Ad-b-gal). Right panel corresponds to Kaplan-Meier survival curve of the tumor growth graft. (0.38 MB PDF) [file pone.0005119.s006.pdf]

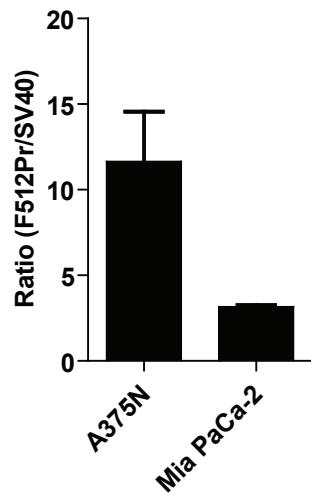

Supplement: Figure S7 — Luciferase activity driven by F512Pr in melanoma and pancreatic cancer cells. Transcriptional activity of F512Pr promoter in MIA PaCa-2 and A375N cells following infection with Ad(I)-F512-luc/Ad-CMV-Renilla. Data are expressed relative to Ad-SV40-luc activity in each cell line. (0.26 MB PDF) [file pone.0005119.s007.pdf]

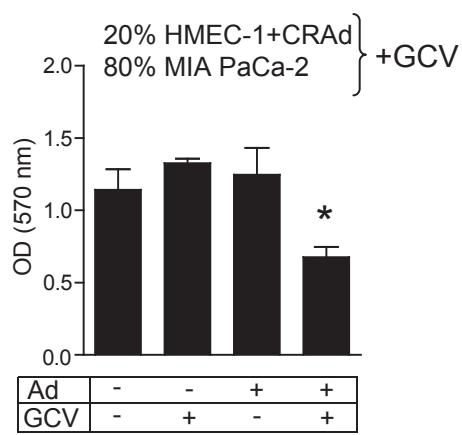

Supplement: Figure S8 — Viability of co-cultures of HMEC-1 cells infected ex vivo with Ad(I)-F512-TK- and human MIA PaCa-2 cells followed by GCV. Only the 20∶80 ratio (HMEC-1:MIAPaCa-2) is shown. *P<0.05 (one-way ANOVA followed by a Tukey multiple comparison test). (0.27 MB PDF) [file pone.0005119.s008.pdf]
