# Supplementary material for: Tumor Associated Stromal Cells Play a Critical Role on the Outcome of the Oncolytic Efficacy of Conditionally Replicative Adenoviruses
Source: PLoS One. 2009 Apr 8;4(4):e5119. doi: 10.1371/journal.pone.0005119 (PMC2663040; doi:10.1371/journal.pone.0005119)

A CRAd Activity+Conditioned medium of stromal cells (serial dilutions)

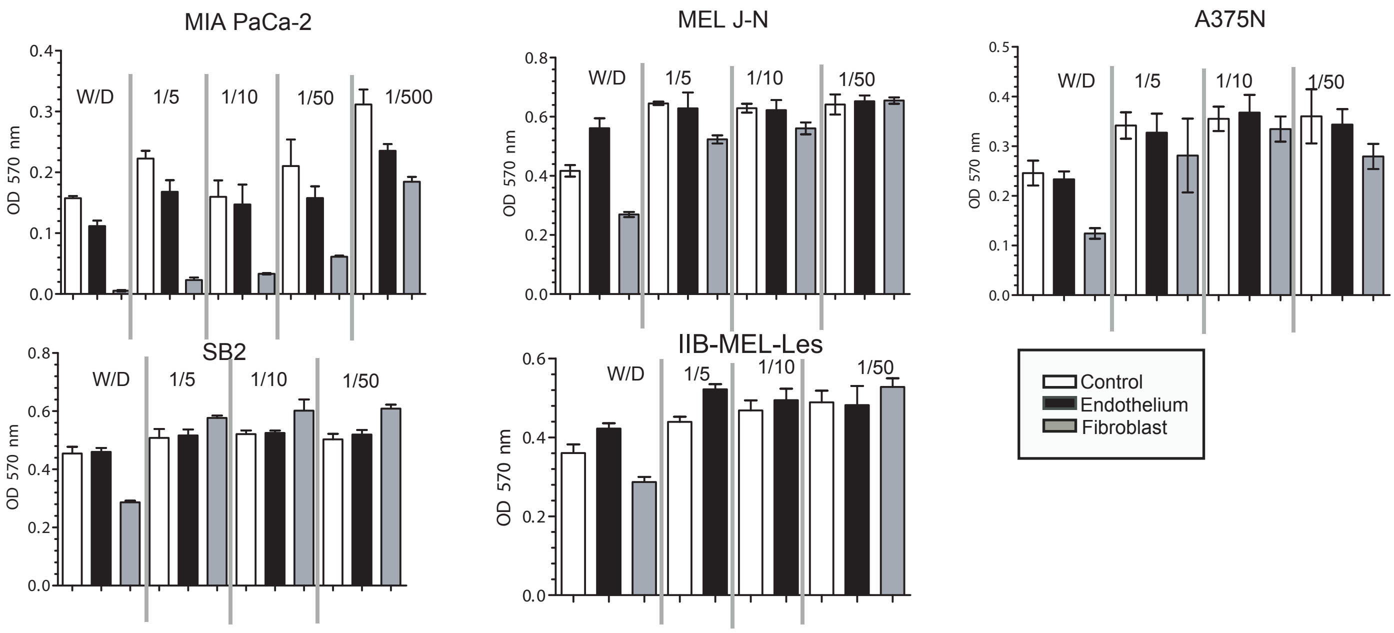

B CRAd Activity+Conditioned medium of infected stromal cells

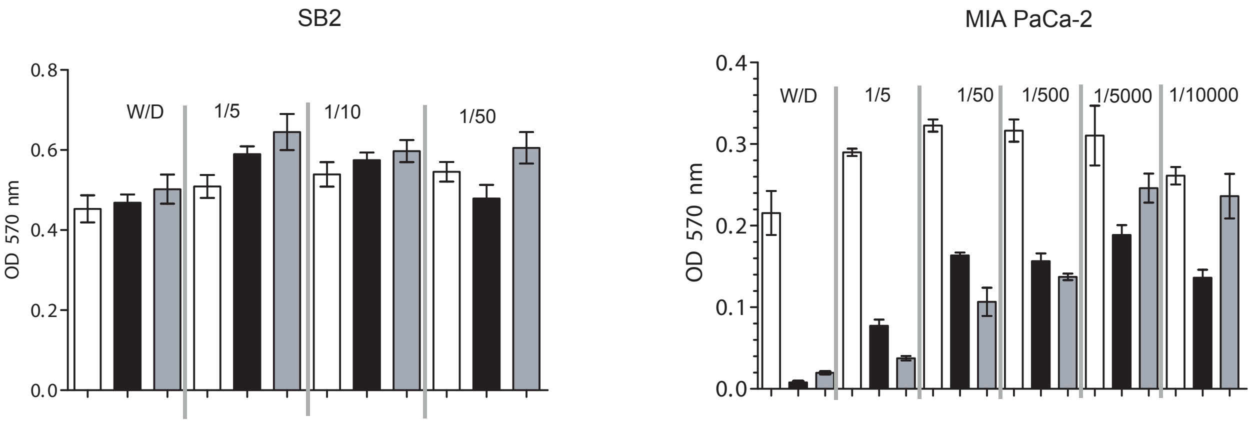

Supplement: Figure S9 — Effect of conditioned media produced by stromal cells on CRAd activity. (A) Cell viability after infection of the different cell types with Ad(I)F512-TK in the presence of different dilutions of conditioned media obtained from HMEC-1 cells, WI-38 fibroblasts or their own (B) Similar to A, but the conditioned media was obtained from pre-infected cells. For further details see Figure 7. Cell viability was assessed at day 6 of infection by using MTT assay. (0.37 MB PDF) [file pone.0005119.s009.pdf]
