# Supplementary material for: Tumor Associated Stromal Cells Play a Critical Role on the Outcome of the Oncolytic Efficacy of Conditionally Replicative Adenoviruses
Source: PLoS One. 2009 Apr 8;4(4):e5119. doi: 10.1371/journal.pone.0005119 (PMC2663040; doi:10.1371/journal.pone.0005119)
Supplement: Table S1 — Primers sequences. The table shows the sequence of primers used for the different clonings. (0.04 MB DOC) [file pone.0005119.s010.doc]

**Table S1: Primers sequences.** The table shows the sequence of primers used for the different clonings.

| **Primer** | **Sequence 5´-3´** |
| --- | --- |
| SPfse | CTAGCTAGCAGCTGGGTGTTGTGGCAT |
| F120 | GaACGCGTgggagaaggaggag |
| F513 | CGACGCGTGCAGCTTGTCTTGTC |
| R35 | CGAGATCTGCTCTCCGGGCAG |
| R28 | CGAGATCTGGGCAGTCTGAAGGACC |
| R71 | GCAGATCTCCTCAGTGGCAGGC |
| R24 | GCAGATCTAGTCTGAAGGACCGCG |
| SPas | ACGCGTCGACCTCAGTGGCAGGCA |
| RTK | GCGTCGACTCAGTTAGCCTCCCCCATCTC |
| FTK | gcCCatggcttcgtaccccggcc |
| INSU-F-SpeI | CCactagtGCTAGAGCTCGCTGATCAGC |
| INSU-R-KpnI | CGGTACCATCCCCAGCATGCCTGC |
| F-E1A-560 | cgAGATCTCcgggactgaaaatgagacat |
| R-E1A-1632 | GCGGATCCAAACATTATCTCACCCTT |
| E4 sense | ACAAGCTCCTCCCGCGTT |
| E4 anti-sense | ACTACGTCCGGCGTTCCAT |
